# Effects of Chronic Fluoxetine Treatment on Neurogenesis and Tryptophan Hydroxylase Expression in Adolescent and Adult Rats

**DOI:** 10.1371/journal.pone.0097603

**Published:** 2014-05-14

**Authors:** Anne Klomp, Lena Václavů, Gideon F. Meerhoff, Liesbeth Reneman, Paul J. Lucassen

**Affiliations:** 1 Swammerdam Institute of Life Sciences, Centre for Neuroscience, University of Amsterdam, Amsterdam, The Netherlands; 2 Department of Radiology, Academic Medical Centre, University of Amsterdam, Amsterdam, The Netherlands; Inserm, France

## Abstract

The antidepressant drug fluoxetine (Prozac) has been increasingly prescribed to children and adolescents with depressive disorders despite a lack of thorough understanding of its therapeutic effects in the paediatric population and of its putative neurodevelopmental effects. Within the framework of PRIOMEDCHILD ERA-NET, we investigated; a) effects of chronic fluoxetine treatment on adult hippocampal neurogenesis, a structural readout relevant for antidepressant action and hippocampal development; b) effects on tryptophan hydroxylase (TPH) expression, a measure of serotonin synthesis; c) whether treatment effects during adolescence differed from treatment at an adult age, and d) whether they were subregion-specific. Stereological quantification of the number of proliferating (Ki-67+) cells and of the number of young migratory neurons (doublecortin+), revealed a significant age-by-treatment interaction effect, indicating that fluoxetine affects both proliferation and neurogenesis in adolescent-treated rats differently than it does in adult-treated rats. In terms of subregional differences, fluoxetine enhanced proliferation mainly in the dorsal parts of the hippocampus, and neurogenesis in both the suprapyramidal and infrapyramidal blades of the dentate gyrus in adolescent-treated rats, while no such differences were seen in adult-treated rats. Fluoxetine exerted similar age-by-treatment interaction effects on TPH cells mainly in the ventral portion of the dorsal raphe nucleus. We conclude that fluoxetine exerts divergent effects on structural plasticity and serotonin synthesis in adolescent versus adult-treated rats. These preliminary data indicate a differential sensitivity of the adolescent brain to this drug and thus warrant further research into their behavioural and translational aspects. Together with recent related findings, they further call for caution in prescribing these drugs to the adolescent population.

## Introduction

Major depressive disorder (MDD) is an intractable mental disorder with a lifetime prevalence of 10–20% in adult life, but childhood and especially adolescent forms of depression are also common [1]. In fact, the early onset forms of depression are associated with a more chronic and severe nature than adult onset depression [2].

The SSRI fluoxetine (FLX; Prozac) is currently the only approved drug available for treatment of paediatric depression, despite ongoing debate over its efficacy in this age category [3], [4] and concerns of increased risk for suicidal thinking in the paediatric population [5]. SSRIs block the 5-HT transporter (SERT) and thereby increase levels of 5-HT in the synaptic cleft. Upon chronic SSRI treatment, 5-HT receptor desensitisation is believed to be instrumental in alleviating depressive symptoms [6], [7]. Tryptophan hydroxylase (TPH) is the rate-limiting enzyme in the synthesis of 5-HT, and the neuronal form, TPH2, is predominantly expressed in the dorsal raphe nucleus (DRN) of the midbrain from where 5-HT projections extend to various brain regions. Its mRNA levels have been found to be increased in post-mortem tissue of non-medicated suicide victims, implicating its involvement in a possible stimulatory response to compensate for low 5-HT levels in depression [8], although depression has not been proven to be solely attributable to reductions in 5-HT *per se* [9].

The neurogenesis theory of depression is a more recent concept that builds on rodent data which show that chronic stress not only induces depressive-like symptoms in animals, but also decreases the incorporation and/or survival of new-born cells in the hippocampus [10], [11], which may relate to the volume reductions found in this brain structure in depression [12]. Furthermore, treatment with FLX can stimulate or rescue neurogenesis in naive [13]–[15] or chronically stressed adult rats, respectively [16], [17]. Neurogenesis is further needed for antidepressants such as FLX and amitriptyline to exert their behavioural effects [18], which are influenced by earlier stress exposure and age [19]–[22]. Furthermore, FLX modulates structural plasticity in human brain [23], [24] but does not alleviate depressive symptoms until after several weeks of treatment, a time-to-effect that matches the time required for new cells to integrate into a neuronal network, which further supports a role for neurogenesis in antidepressant action [24].

Recently, we found differential, age-dependent effects of FLX treatment on 5-HT related brain activity using pharmacological MRI [25]. This suggests that in terms of sensitivity to FLX, the brain of adolescent animals differs from that of adults, which may bear relevance for ongoing developmental processes [26]. In view of the ongoing development of 5-HT transmission in adolescence and the fact that 5-HT influences neurogenesis [27], [28], exposure to SSRIs during dentate gyrus (DG) development may exert lasting consequences on the adult 5-HT system, and influence later vulnerability to psychopathology [27]. Despite the wealth of studies investigating the effects of maternal FLX exposure on neurogenesis, there is a paucity of studies on adolescent exposure and those that exist offer mixed results [29]–[31].

Tryptophan hydroxylase (TPH) expression reflects the rate of 5-HT synthesis. Further, chronic SSRI treatment reduced TPH levels after adult exposure and after treatment during the early postnatal period [32], [33]. In addition, FLX treatment has been indicated to influence 5-HT in the midbrain raphe nucleus, where TPH is abundant, and to mediate antidepressant effects [34]. The differential and age-dependent effects of SSRIs on neurogenesis have been alluded to before [35]. Since adolescence represents a sensitive developmental period, environmental factors such as stress or psychotropic drugs can influence the maturation of various important brain circuits, often in a lasting manner [2], [26]. Since little is still known, however, on the effects of chronic SSRI exposure on TPH expression and on neurogenesis markers during adolescence, we here set out to determine whether chronic FLX treatment induces age-dependent and regional-specific effects on proliferation, adult neurogenesis and TPH expression, and notably, whether interaction effects exist.

## Materials and Methods

All experiments were carried out according to the guidelines set forth by Dutch regulations on animal welfare and protection. Protocols were reviewed and approved by the local Animal Welfare Committee for Animal Experiments at the Academic Medical Centre in Amsterdam, the Netherlands. All efforts were made to ensure that animal suffering and the number of animals used was minimal.

### Animals

In total, 32 male Wistar rats (n = 8 per group) were obtained from Harlan (Venray, The Netherlands). The adolescent-treated animals arrived on postnatal day 21 (PND21) and treatment started at PND25 (body weight of 50–80 g) with either FLX or saline vehicle (SAL). Since the peripubescent period begins around PND24 [36], PND25 lent itself to model the clinical situation in which FLX is administered to children and adolescents from the age of 8 years onwards. A second group of young adult animals began treatment at PND65±4 days (body weight of 290–320 g). Henceforth, the group of rats treated in adolescence will be referred to as PND25 and the group treated in adulthood as PND65. All animals were group-housed, four per cage, weighed and handled daily and provided with food and water *ad libitum*. A normal 12∶12 hour light/dark cycle was applied with lights on at 7:00 *am*. Laboratory conditions were kept at 20 ± 1°C with normal humidity.

### Drugs and treatment protocol

**Figure 1:**
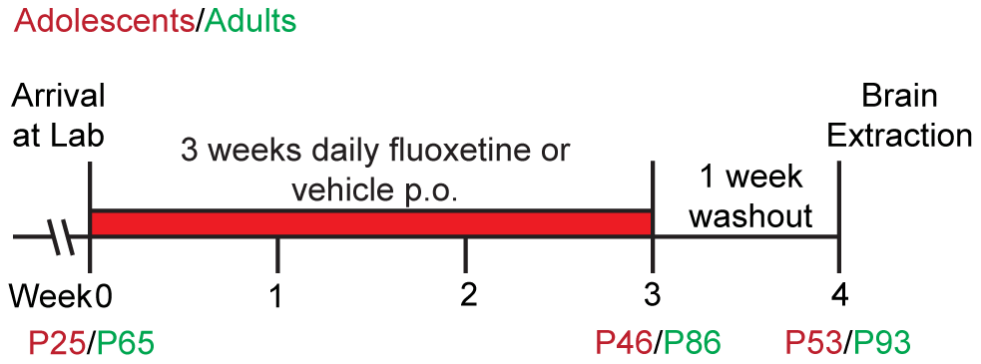
Chronological timeline representing the experimental protocol. Two age groups were used: the adolescent-treated group (referred to as P25) and the adult-treated group (referred to as P67). After an acclimatisation period of 4 days, chronic treatment with either FLX (5 mg/kg) or vehicle started at the age of P25 or P67. After 21 days of treatment and one week of drug washout, adolescent and adult brains were extracted at P53 or P95 respectively.

Fluoxetine hydrochloride (Fagron, Belgium) was dissolved in sterile water (vehicle) and administered at a final dose of 5 mg/kg/day via oral gavage with a total volume of 0.5 ml in young animals and 1 ml in adult animals. Control animals received the same total volumes of vehicle by oral gavage. Treatments were administered daily before lights out for 3 weeks, followed by a washout period of one week to ensure the pharmacological agent had left the animals' system. Also see **Figure 1** for the time line of this study.

### Tissue Preparation

At the end of the washout period, animals were sacrificed at PND53 in the adolescent group, and at PND93±4 in the adult group. Animals were anaesthetised with sodium pentobarbital and transcardially perfused with 0.9% saline followed by 4% paraformaldehyde in 0.1 M Phosphate Buffered Saline (PBS), pH 7.4. Brains were post-fixed in the skull overnight at 4°C before careful removal and storage in PBS azide pH 7.4. Subsequently, brains were cryoprotected by saturation in 15% sucrose, later replaced by a 30% sucrose solution in PB. Sections were cut at 40 µm with a freezing microtome. Tissue sections were collected in a one-in-10 series in 0.1 M phosphate buffer and ranged from the prefrontal cortex (Bregma 5.2) through to the dorsal raphe nucleus (Bregma −10.30, [37]). Before sectioning, a small incision was made in the left hemisphere to identify left/right differences. Sections were washed in chilled PB to extract the cryoprotectant and stored at 4°C in PB + 0.01% sodium azide prior to immunohistochemistry.

### Immunohistochemistry

To study changes in proliferation in the adult brain, Ki-67 immunohistochemistry was used. Ki-67 is an endogenous protein expressed during all stages of the cell cycle, except G_0_. Free-floating coronal sections were mounted on glass slides, dried and randomised, placed in plastic containers, and further processed according to protocols described previously [38] with initial antigen retrieval steps in boiling citrate buffer (0.01 M, pH 6.0) and endogenous peroxidase blocking before primary antibody application with Ki-67 (polyclonal rabbit anti-Ki-67p; 1∶2000; Novocastra) and secondary antibody biotinylated goat anti-rabbit (Vector, 1∶200), and finally amplification with an avidin-biotin complex (ABC-Elite; 1∶800 in PBS) before chromogenesis with the peroxidase substrate 3,3-“-Diaminobenzidine tetra-hydrochloride (DAB).

To determine if FLX had influenced survival and neuronal differentiation of progenitor cells, immunocytochemistry was also performed for doublecortin (DCX), a microtubule-associated protein expressed in young migratory neurons, commonly used as an indicator of neurogenesis. Free-floating hippocampal sections were processed according to protocols described previously [38] by blocking endogenous peroxidase with 0.5% H_2_O_2_ in TBS and 2% milk powder to prevent non-specific binding before anti-doublecortin (Santa-Cruz 1∶800) and biotinylated donkey anti-goat (Jackson, 1∶500) were applied and developed with ABC-Elite and DAB.

Tryptophan hydroxylase 2 (TPH) was studied, as it is the classical rate-limiting enzyme of 5-HT synthesis in the brain. Free-floating sections of the dorsal raphe nuclei (DRN) were used. Following initial block steps for endogenous peroxidase, 5% Normal Rabbit Serum with 0.2% Triton-X 100 in PBS was applied and finally the primary antibody (sheep anti-Tryptophan Hydroxylase 2; 1∶1000; Chemicon/Millipore, AB1541) was dissolved in 1% Normal Rabbit Serum with 0.2% Triton-X 100 in PBS. The secondary antibody was biotinylated Rabbit Anti-Sheep (Vector; 1∶200). Signal was amplified with ABC-Elite (1∶800 in PBS) and developed with DAB.

### Data acquisition and stereological quantification

**Figure 2:**
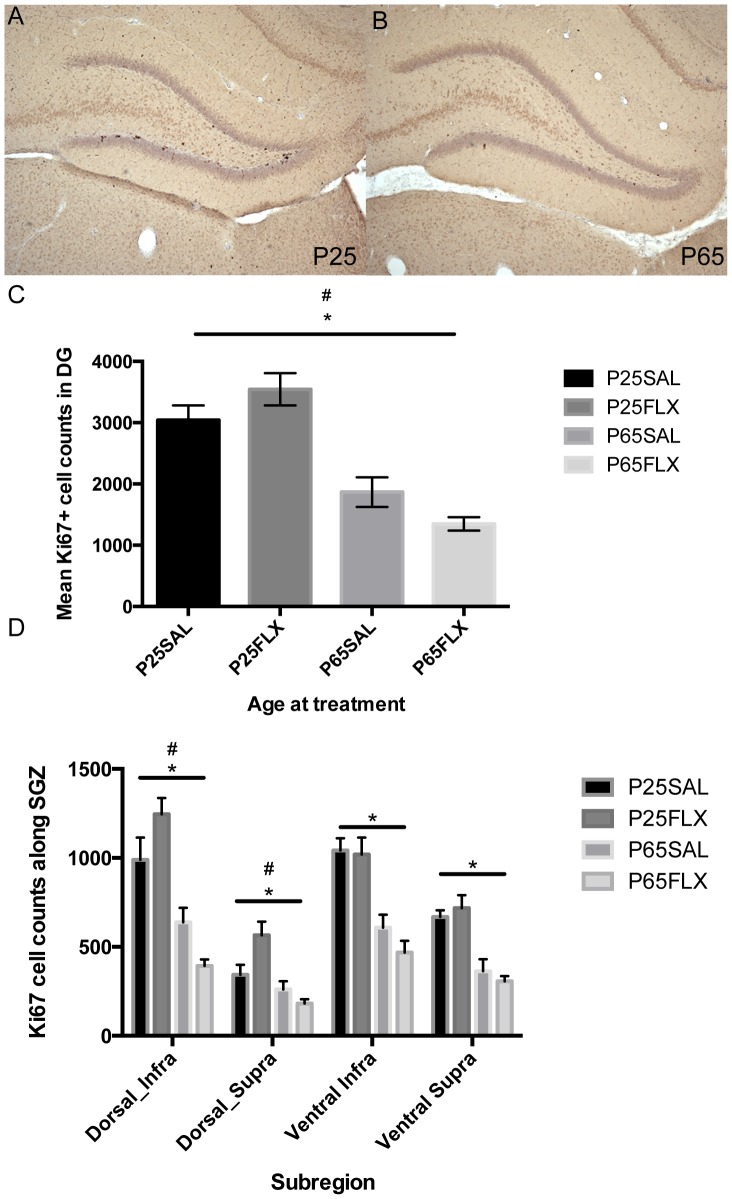
Age-related effects of fluoxetine and regional differences in cell proliferation. An example of Ki-67 expression in the dentate gyrus of the hippocampus is shown for (**A**) adolescent-treated and (**B**) adult-treated rats. **C**) Two-Way ANOVA revealed a significant age-by-treatment interaction effect (p  =  0.029) and a significant effect of age (p < 0.001) in the expression of Ki-67+ cells; **D**) Sub-Regional differences in proliferation were present in the hippocampus (divided into the infrapyramidal and suprapyramidal blades of the dentate gyrus in either the dorsal or ventral portion of the hippocampus). There was a significant effect of age in all sub-regions (p < 0.001), and a significant age-by-treatment interaction effect in both the dorsal infrapyramidal (p  =  0.009) and the dorsal suprapyramidal blade (p  =  0.017). Also note the higher cell numbers in the infrapyramidal blade overall compared with the suprapyramidal blade, regardless of the dorsal or ventral portion. *  =  main effect of age; #  =  age-by-treatment effect. P-values below 0.050 were considered statistically significant. Error bars indicate ± 1 S.E.M.

Ki-67+ cells in the DG were quantified in the granule cell layer (GCL), subgranular zone SGZ) and hilus of the dentate gyrus by an observer (LV) unaware of the conditions of the material, using a light microscope (**Figure 2A and B**). The SGZ was defined as a three cell-body wide band running along the base of the GCL facing the hilus. The hilus was defined by drawing a virtual line from the caudal tip of the suprapyramidal blade to the tip of the CA3-4 that ended within the DG, and then to the caudal end of the infrapyramidal blade. Ki-67+ cells were quantified stereologically in a 1-in-10 series (11 ±1 hippocampal sections per animal) in the SGZ of the infrapyramidal and suprapyramidal blade, in the GCL of both infra- and supra-pyramidal blades, and in the hilus, in both hemispheres. Cell counts were multiplied by 10 to express total estimated cell numbers for all neurogenesis parameters. Dorsal and ventral regions were defined as the first 6 and final 4–6 hippocampal sections, respectively, along the entire extent of the rostro-caudal axis.

**Figure 3:**
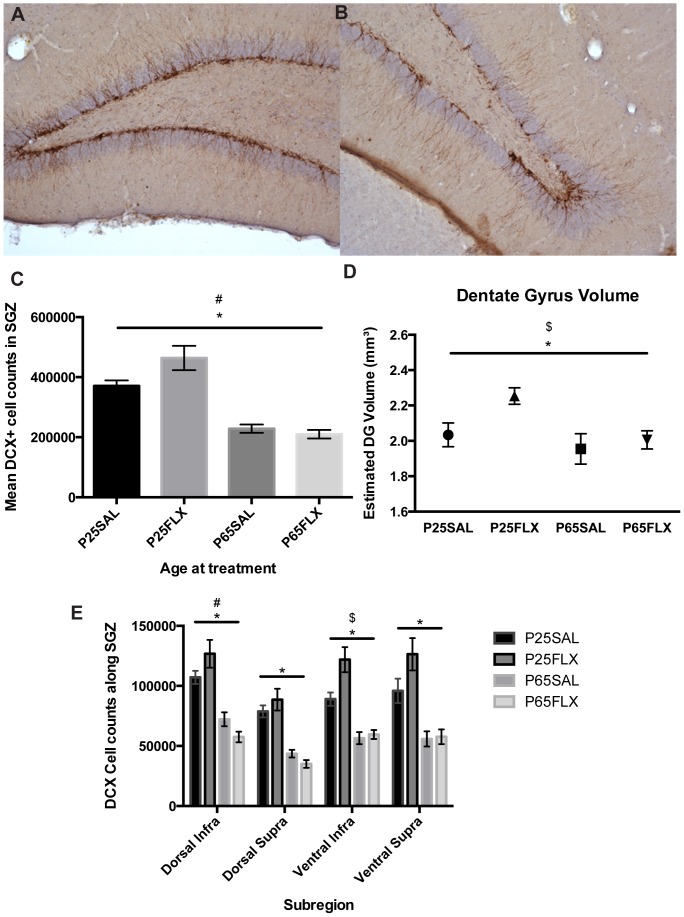
Age-related effects of fluoxetine and regional differences in cell differentiation. An example of doublecortin (DCX) expression along the subgranular zone is shown for adult-treated (**A**) and adolescent-treated rats (**B**). **C**) Two-Way ANOVA revealed both a significant age-by-treatment interaction effect (p  =  0.036) and a significant effect of age (p < 0.001) on the expression of DCX+ cells. **D**) There were regional differences in the amount of DCX+ cells. There was a significant effect of age in all sub-regions (p < 0.001), a significant treatment effect in the ventral infrapyramidal blade of the dentate gyrus (p  =  0.021), and a significant age-by-treatment interaction effect in the dorsal infrapyramidal blade (p  =  0.028). **E**) There was both a significant effect of age (p  =  0.017) as well as a main effect of treatment (p  =  0.045) on dentate gyrus volume. Dentate gyrus volume comprised the SGZ plus GCL. *  =  main effect of age; 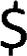  =  main effect of treatment; #  =  age-by-treatment effect. P-values below 0.050 were considered statistically significant. Error bars indicate ± 1 S.E.M.

DCX+ cells were too numerous to be counted by hand (**Figure 3A and B**), and a design-based stereological procedure was therefore applied based on every tenth serial section along the rostro-caudal extent of the hippocampus (12 sections per animal) as described before [38] including the infra- and supra-pyramidal blades as well as the dorsal and ventral hippocampus as described above. DCX+ cells were quantified using a systematic random sampling technique with an optical fractionator method using the StereoInvestigator software (MicroBrightField, Germany). The following settings were employed: grid size 140×80, counting frame 50×50, which resulted in counts ranging from 300 to 800. Cells were counted at 40× magnification using a Zeiss microscope coupled to the StereoInvestigator software.

**Figure 4:**
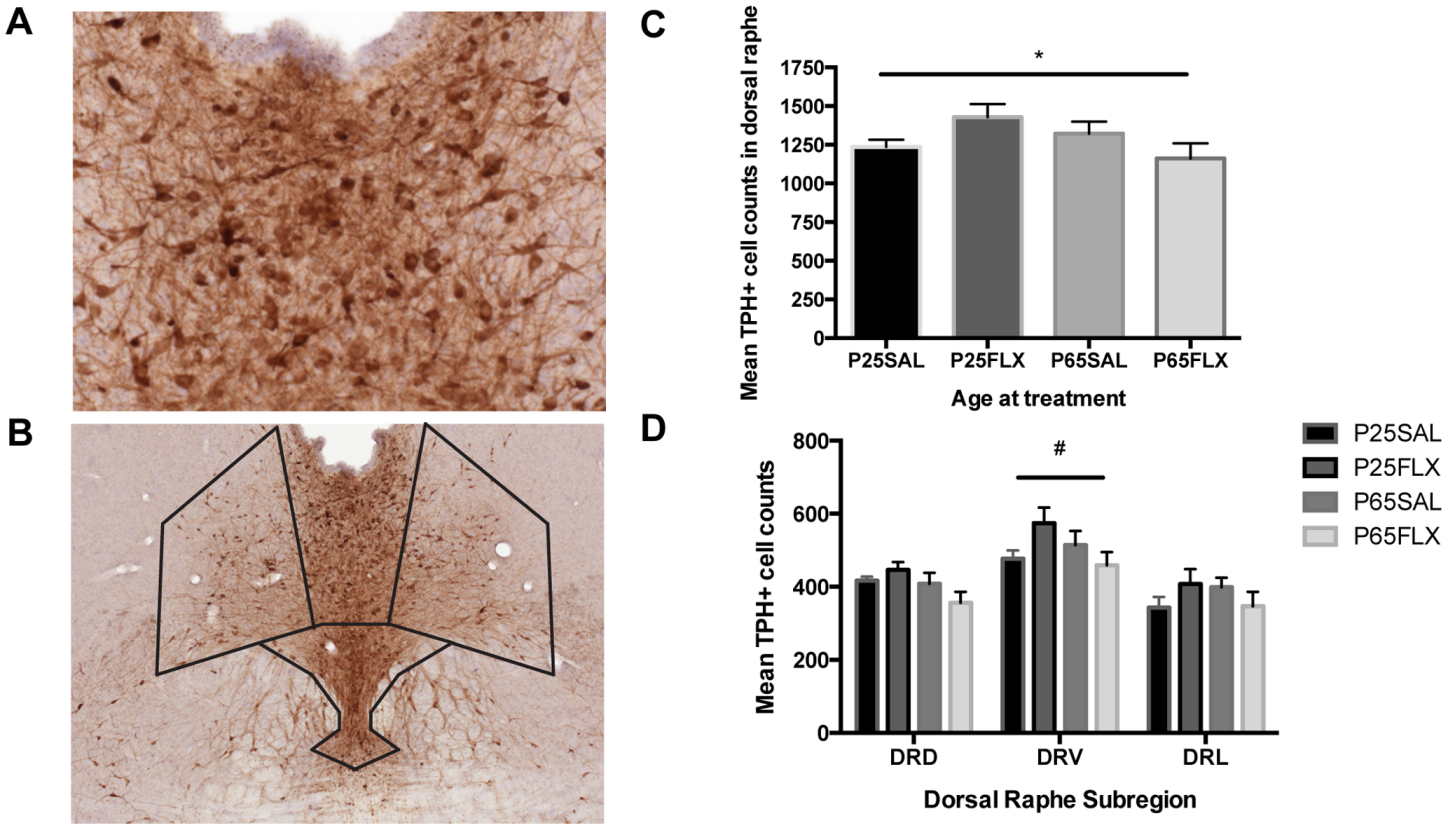
Age-related effects of fluoxetine and regional differences in serotonin synthesis. **A**) Example of TPH expression in the entire DRN and **B**) in the dorsal portion of the DRN. **C**) There was a significant age-by-treatment interaction effect (p  =  0.033) on TPH+ cells in the total DRN (indicated by the line and asteriks above the four bars). **D**) Two-Way ANOVA on regional differences showed a significant age-by-treatment interaction effect (p  =  0.043) in the DRV only. *  =  main effect of age; 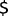  =  main effect of treatment; #  =  age-by-treatment effect. P-values below 0.050 were considered statistically significant. Error bars indicate ± 1 S.E.M.

TPH+ cell numbers were quantified in the dorsal raphe at 20× magnification in every 10^th^ section (See **Figure 4A** for an example). The dorsal raphe was divided into sub-regions, believed to support different functional roles with respect to serotonergic projections; i.e. the dorsal raphe dorsal (DRD), dorsal raphe ventral (DRV) and dorsal raphe lateral (DRL) nuclei, which comprise two wings lateral to the DRD that both join the DRV (see **Figure 4B**).

### Statistical analyses

All data are expressed as group means ± S.E.M. Data were analysed using SPSS.20 (IBM) by analysis of variance (Two-Way ANOVA) with ‘age’ and ‘treatment’ as factors, after log-transformation of the data where necessary. In the existence of significant interaction effects, bonferroni post-hoc analyses were performed to elucidate the differences between groups within conditions. A paired Student's *t*-test was used to evaluate interhemispheric differences. P-values of ≤ 0.05 were considered statistically significant.

## Results

### Cell Proliferation

Quantification of Ki-67+ cell numbers in the bilateral SGZ, GCL and hilus revealed no significant differences between left and right hemispheres in any group (P65SAL: *t*(4)  =  0.456, *p* = 0.672; P65FLX: *t*(4)  =  0.706, *p* = 0.519; P25SAL: *t*(6)  = −0.721, *p* = 0.498; and P25FLX: *t*(6) = 1.801, *p* = 0.122, paired student's *t*-test). In the SGZ, a significant main effect of age was found (Two-Way ANOVA; F(1,28)  =  57.140, *p*<0.001) regardless of FLX treatment, as well as a significant age-by-treatment interaction effect (F(1,28)  =  5.286, *p* = 0.029), indicating that FLX exerts a different effect on cell proliferation depending on age-at-treatment (**Figure 2C**). Bonferroni post-hoc analysis showed no significant FLX-induced reduction in cell proliferation in adult-treated rats, neither did it indicate a significant FLX-induced increase in adolescent-treated rats. There was also a significant age-by-treatment interaction effect detected in the hilus (F(1,28) = 8.122, *p* = 0.008) as well as a significant main effect of age (F(1,28) = 58.316, *p*<0.001) reflecting the overall effects seen on proliferation in the SGZ.

The infrapyramidal blade of the dorsal hippocampus (dorsal infra), the suprapyramidal blade of the dorsal hippocampus (dorsal supra) and those of the ventral hippocampus (ventral infra and ventral supra) were analysed separately. The highest numbers of Ki-67+ cells were observed in the infrapyramidal blade of all groups, relative to the suprapyramidal blade (**Figure 2D**). A Two-Way ANOVA revealed significant age effects, irrespective of treatment, in all subregions (dorsal infra: F(1,28)  =  45.041, *p*<0.001; dorsal supra: F(1,28)  =  18.197, *p*<0.001; ventral infra: F(1,28)  =  40.77, *p*<0.001; ventral supra: F(1,28)  =  41.414, *p*<0.001). Furthermore, there was a significant age-by-treatment interaction in the dorsal infra (F(1,28)  =  7.810, *p* = 0.009) and in the dorsal suprapyramidal blade (F(1,28)  =  6.434, *p* = 0.017). Bonferroni post-hoc analysis of the dorsal hippocampus however, revealed no significant changes induced by FLX treatment in either group in the dorsal infra, nor in the dorsal supra. Group comparisons in the dorsal and ventral hippocampus revealed significant main effects of age in both the dorsal F(1,28) = 39.252, *p*<0.001, and ventral F(1,28) = 59.043, *p*<0.001 hippocampus and a significant age-by-treatment interaction effect in the dorsal hippocampus F(1,28) = 9.064, p = 0.005. Together this suggests that FLX primarily exerts effects, although subtle, in the dorsal hippocampus, and that the direction of the effects of FLX on cell proliferation is largely driven by and dependent on the age-at-treatment.

### Neurogenesis

Stereological quantification of DCX+ (doublecortin-positive) cell numbers revealed a significant main effect of age, irrespective of treatment, when the DG was taken as a whole (Two-Way ANOVA F(1,28)  =  92.172, *p*<0.001) and a significant age-by-treatment interaction effect (F(1,28)  =  4.852, *p* = 0.036), such that adolescent-treated animals had higher numbers of DCX+ cells and adult-treated animals lower numbers of DCX+ cells (**Figure 3C**), indicating that FLX had an age-dependent, divergent effect on neuronal differentiation. Bonferroni post-hoc analyses revealed no significant FLX-induced changes on adult-treated (p = 1.000) nor adolescent-treated animals (p = 0.227).

When investigating regional effects of FLX, a Two-Way ANOVA revealed significant age effects, irrespective of treatment, in all sub-regions (dorsal infra: F(1,28)  =  55.837, *p*<0.001; dorsal supra: F(1,28)  =  80.664, *p*<0.001; ventral infra: F(1,28)  =  61.084, *p*<0.001; and ventral supra: F(1,28)  =  40.455, *p*<0.001), indicating an age-dependent decline in cell differentiation. Additionally, a significant main effect of treatment was detected in the ventral infra subregion (F(1,28)  =  5.969, *p* = 0.021) such that FLX increased DCX+ cells in adolescents only (Bonferroni post-hoc; p = 0.047). Furthermore, there was a significant age-by-treatment interaction effect in the dorsal infra (F(1,28)  =  5.369, *p* = 0.028) such that FLX age-dependently influenced DCX+ cells (**Figure 3D**), however Bonferroni post-hoc did not show significant FLX-induced changes in adult-treated (p = 0.341) nor adolescent-treated animals (p = 1.000). Group comparisons in the dorsal and ventral hippocampus displayed significant age effects (*p*<0.001) in both the dorsal and ventral regions and a significant main effect of treatment in the ventral hippocampus F(1,28) = 5.363, *p* = 0.028, such that FLX increased neurogenesis in P25-treated animals only (Bonferroni post-hoc *p* = 0.024).Together, these results indicate that FLX exerts its effects on neuronal differentiation primarily in the infrapyramidal blade of the ventral hippocampus in adolescent-treated animals.

Analysis of DG volume (SGZ and GCL) divulged an age effect (F(1,28)  =  6.461, *p* = 0.017) with adolescents having slightly but significantly larger DG volumes than adults, and a significant treatment effect (F(1,28)  =  4.418, *p* = 0.045) such that FLX increased the total volume of the DG independent of age (**Figure 3E**).

### Tryptophan hydroxylase

A significant age-by-treatment interaction effect was revealed when the DRN was taken as a whole (Two-Way ANOVA F(1,28)  =  5.030, *p* = 0.033), suggesting FLX increased TPH immunoreactivity in adolescent-treated animals and reduced TPH immunoreactivity in adult-treated animals (**Figure 4C**). Bonferroni post-hoc analysis showed no significant age differences in non-treated animals (p = 1.000) or treatment differences in adolescent (p = 0.567) or adult-treated (p = 0.964) rats indicating the interaction effect was driven purely by the difference in TPH levels after FLX. Thus, as a whole, TPH expression in the DRN was age-dependently influenced by FLX. When the dorsal raphe was subdivided into dorsal raphe dorsal (DRD), dorsal raphe ventral (DRV) and dorsal raphe lateral (DRL), there were no significant treatment or interaction effects in the DRD, although a borderline significant main effect of age was present (F(1,28)  =  4.153, *p* = 0.051). In the DRV, there was a significant age-by-treatment interaction effect (F(1,28)  =  4.500, *p* = 0.043) such that FLX influenced the number of TPH+ cells differently, depending on age (**Figure 4D**). Bonferroni post-hoc of the DRV showed no clear FLX-induced changes in either adolescent (p = 0.398) or adult-treated (p = 1.000) animals. Together, the results from the TPH immunohistochemistry indicate that FLX exerts its effects mainly in the ventral portion of the DRN.

## Discussion

The present study investigated age- and region-specific changes in structural plasticity and TPH expression after chronic FLX treatment, administered either during adolescence or adulthood. One week after a 3-week period of chronic FLX exposure, we observed a significant effect on neurogenesis and proliferation parameters, which were dependent on age-at-treatment. Adolescent exposure to FLX, but not adult exposure, significantly increased neurogenesis in the ventral hippocampus. Likewise, the differential effects of FLX on the number of TPH+ cells in the ventral portion of the DRN were also dependent on age-at-treatment, where adolescent treated rats had higher TPH+ expression than adult treated rats.

Adult neurogenesis is a commonly used measure for structural plasticity in rodents [24]. It is extensive in the early life period and slows down with age [39], a finding consistent with the significant age effects reported here. In young rats, the DG is formed during the first 2–3 weeks of life and approximately 80% of its granule neurons are born postnatal. In rat, adolescence lasts from PND28 to PND60, with puberty occurring around PND45 [36]. Hence, our experimental design in which FLX treatment was started at PND25 and lasted for 3 weeks, not only approximated adolescence in humans, but also coincided with the sensitive period of delayed DG development in the rat. As such, it was optimally suited to detect putative effects on structural organisation in a particular temporal window.

In addition to timing, the dose of the drug is important. For human depression, the most effective dose of FLX is 20 mg (approx. 0.3–0.9 mg/kg) [40]. In rats, however, drugs have generally been administered at 10-fold higher doses because of the difference in the rate of liver metabolism and hence a clinically relevant dose of FLX is 3–9 mg/kg. A FLX dose of 5 mg/kg is commonly used in rat studies and is sufficient to induce blockade of 5-HT and inhibition of SERT [41], [42]. Despite this, most rodent studies have used higher FLX concentrations, typically 10–20 mg/kg, which are not only clinically less relevant, but also induce behavioural disturbances including impairments in water-maze probe trial performance and/or anxiety after doses of FLX up to 10 mg/kg/day from PND28-60 [17], [43]. Moreover, since behaviour was not investigated in the present study, a dose of 5 mg/kg was opted for, particularly since higher FLX doses have previously been shown to affect motor function, which in turn can starkly modify neurogenesis [44].

### Effects of adult FLX exposure on neurogenesis

Even though neurogenesis is reduced with age, it can still be stimulated by environmental factors, or in depression (models) with various antidepressants [12], [45]–[47] including FLX, which stimulates proliferation in young, naive rodents [13] but not in older animals [20], [29], [44]. The present findings which lean towards a reduction in proliferation and DCX+ numbers in adult-treated animals differ from these earlier, mostly stimulatory, reports on FLX [18], [45], [48]. However, these initial studies employed different experimental designs, different markers for structural plasticity and/or studied considerably shorter survival times. Moreover, several groups have now demonstrated that the stimulatory effects of antidepressants like FLX on neurogenesis parameters, strongly depend on the strain, age and brain region of the animal [18], [49] and on its stress history [20]–[22], [44], [50], [51]. Consistent with our present results, several studies failed to find stimulatory effects of FLX or other antidepressants on neurogenesis in rodents of comparable ages, or they have reported decreases in neurogenesis [16], [29], [31], [46], [52] or neurogenesis-independent effects [21], [53].

Another difference with these previous studies is the washout period of 1 week that we introduced to avoid acute effects of the drug on neurogenesis and TPH readouts. Although this design enabled us to study lasting effects, it could also have allowed compensatory responses to develop, which may have crucially masked initial differences between groups by the time of sacrifice. Similar dynamic responses are known from studies in which neurogenesis was initially reduced by radiation, chemotherapy or stress, but later showed recovery [54], [55]. The current results can then be explained by assuming that neurogenesis is stimulated by chronic FLX treatment, but later reduced when the drug is no longer present. If this is true, it means the drug should remain continuously present in order to propagate its positive effect on neurogenesis at this age. Another option is that exhaustion, or depletion, of the initial neurogenic pool has occurred, which might be due to forced increases in division of the progenitor cells by the early FLX treatment, which may have slowed down subsequent stages of neurogenesis at later time points [15].

### Effects of adolescent FLX exposure on neurogenesis

We found that FLX widened the gap between adolescent-and adult-treated animals in terms of neurogenesis such that adolescents expressed stimulatory effects on neurogenesis compared to their older counterparts. Indeed FLX had a significant stimulatory effect on neurogenesis in the ventral hippocampus in adolescent-treated but not adult-treated animals. This is consistent with an earlier study performed in mice [31] that also reported stimulatory effects of FLX (16 mg/kg/day in drinking water for 14 days) on neurogenesis, but only when treatment was initiated during adolescence and not in adulthood. However, others failed to find effects of adolescent FLX treatment, both acutely and after several weeks of treatment discontinuation [29], [30]. Despite having studied rats of comparable ages and with similar FLX doses, i.e. 5 mg/kg (i.p.), Cowen et al. (2008)[29] reported no effect of FLX whatsoever, whereas Hodes et al., (2009)[30] only found FLX to increase cell proliferation in adult male rats but not in the peri-pubescent male, or female, rats. Also, the treatment duration in our study was comparable to that of Navailles et al. (2008)[31], i.e. between 14 and 25 days.

An explanation of these inconsistent findings might lie in effects of social stress, which is known to inhibit proliferation, in combination with FLX which could potentially reverse reductions in neurogenesis. On the other hand, stimulatory effects of adolescent FLX exposure on neurogenesis can be abolished by early life stress [31]. In both studies mentioned above [29], [30], animals were individually housed just before initiation of the treatment. This presents a serious social stressor for juvenile animals that are particularly sensitive to environmental stress, especially since brain areas targeted by stress such as the prefrontal cortex and hippocampus develop relatively late. In line with this, social play behaviour is disrupted after earlier individual housing [56], and social isolation can even preclude the positive effects of running on adult neurogenesis [57]. It is thus likely that the social isolation just before and during treatment in these earlier studies by Hodes and Cowen may have masked effects of FLX on neurogenesis.

While changes in neurogenesis following a specific stimulus such as FLX are generally transient during adulthood [45], such stimuli occurring during the adolescent period can exert lasting ‘imprinting’ effects on DG development [26] and it would therefore be interesting to study whether the increased numbers of proliferating and differentiating cells in our current study do survive and, in addition to volume, further change DG structure and function at even later ages, as previously demonstrated following early stress exposure [22]. This awaits additional studies.

### Subregional differences in neurogenesis

The present study is the first to investigate effects of FLX on distinct anatomical subregions of the hippocampus in animals of this age. Given the evidence for the functional dissociations between the supra- and infrapyramidal blades and the rostral and caudal subregions of the hippocampus, the location in which adult-generated neurons reside becomes highly relevant. Also, the separate subregions of the hippocampus support distinct functional roles; while spatial processing relies on the dorsal hippocampus, anxiety-related behaviour and possibly also depression aetiology (e.g. [58]) are associated with the ventral hippocampus. In addition, the dorsal hippocampus is innervated primarily by 5-HT projections from the median raphe, whereas the ventral hippocampus is innervated by projections from both the dorsal and median raphe [59]. Within the dorsal and ventral hippocampus, the infra- and suprapyramidal blades of the DG further differ in many anatomical, morphological and molecular aspects, with the suprapyramidal blade e.g. receiving more synaptic connections and preferential input from the entorhinal cortex [60].

During development, neurogenesis proceeds along ventral to dorsal as well as suprapyramidal to infrapyramidal gradients, and although distinct topographical differences remain in the adult hippocampus [61], it was still unclear whether regional differences can be influenced by FLX treatment. Literature suggests that FLX may induce differential effects in the ventral DG and that neurogenesis can be regulated independently in the dorsal and ventral DG respectively [62].

Interestingly, the dorsal infrapyramidal blade has been implicated as the primary region where FLX induces its effects, thereby playing an important role in activating the hippocampus [63]. In the present study, we report that FLX increased cell proliferation in both blades in the adolescent group but only in the dorsal hippocampus, while differentiation was increased throughout the dorsal as well as ventral regions of the hippocampus. This corroborates previous findings which report increased cell survival in the dorsal infrapyramidal blade, i.e. in the region that does not support spatial learning, in response to chronic fluoxetine treatment [62]. These authors did not include the ventral hippocampus, so dorsal/ventral comparisons cannot be made with our study.

We also report a main effect of treatment on cell differentiation in the infrapyramidal blade of the ventral hippocampus in adolescents, the same subregion implicated in anxiogenic behaviour [58]. The literature also implicates distinct anatomical changes in radial glia-like progenitors in this part of the hippocampus [61] and these regional specific effects could explain part of the discrepancies in literature on FLX effects on neurogenesis. For example, stress targets proliferation and neurogenesis preferentially in the ventral hippocampus, an effect reversed by FLX treatment [18], [62]. In contrast, a positive stimulus such as environmental enrichment, increases cell proliferation in both divisions, but only promotes actual neurogenesis in the dorsal hippocampus [59]. These reports are supplemented by the present finding that cell differentiation was increased by FLX treatment in both age groups in the ventral hippocampus in the infrapyramidal blade. Our finding that increased neuronal differentiation was found in the ventral hippocampus of adolescent-treated animals is corroborated by findings in rats [64] and in primates where SSRI or TCA treatment induced increased numbers of cell divisions in the anterior hippocampus [18], [23], the equivalent of the rodent ventral hippocampus, which in humans is connected to the amygdala, prefrontal cortex and nucleus accumbens, regions involved in emotional and reward pathways.

Together, these results indicate that environmental and pharmacological factors seem to exert differential effects on neurogenesis in a region-specific manner, with FLX being preferential to the ventral hippocampus, and non-pharmacological external factors preferential to the dorsal hippocampus. This may underlie separate roles of different populations of neurons along the septo-temporal axis, with specific functional implications for stress-related psychopathologies such as anxiety disorder or depression.

### Possible mechanisms of action underlying the age-dependent effects of FLX on neurogenesis

5-HT plays a key role in many aspects of early brain development, including cell proliferation and differentiation, and is crucial for proper wiring of the brain [28], [65]. In a previous study, we found FLX to increase anxiety-like behaviour in adult but not adolescent rats, which was paralleled by increased SERT densities in most cortical brain regions in the adolescent, but not adult, treated rats [66]. This indicated that effects of FLX on the 5-HT system are age-dependent [67], consistent with the present neurogenic effects in the developing brain that differ from those in the adult.

Although it is still not clear how precisely 5-HT transmission regulates adult neurogenesis [68], FLX is thought to exert its effect by primarily targeting early progenitors in the DG. It remains largely unknown which 5-HT receptors are present on individual progenitor cells, or whether FLX alters their function, or e.g. glucocorticoid hormone receptor or NMDA receptor expression on the new cells. Another important player in the regulation of neurogenesis is BDNF (brain derived neurotrophic factor) since actions of FLX on neurogenesis are believed to depend on BDNF activity [69]. In turn, BDNF expression is thought to be regulated by 5-HT transmission. As suggested by Migliarini et al. (2012)[27], effects of 5-HT depletion on BDNF expression propose a possible regulatory feedback mechanism through which 5-HT itself might regulate the formation of the 5-HTergic neuronal circuitry in the hippocampus. A reduction in 5-HT levels may trigger endogenous BDNF signalling that would then exert a neurotrophic effect on 5-HTergic axons, which in turn, could restore original BDNF levels. Ongoing plasticity within this regulatory feedback loop during adolescence could be accountable for age-dependent effects of chronic FLX on adult neurogenesis.

### Fluoxetine treatment and tryptophan hydroxylase expression

Since TPH is the rate-limiting enzyme in 5-HT synthesis, its immunocytochemically detected protein levels are considered an index of 5-HT synthesis [70]. Consistent with observations that increases in 5-HT release could affect adult neurogenesis [68], the change observed here in TPH+ cells in the DRN corresponded in direction to the change observed in DCX and Ki-67 cell numbers in the DG. The effects of chronic FLX on TPH immunoreactivity are indicative of a clear age-at-treatment dependence, with post-hoc tests showing higher TPH expression in adolescent-treated animals and a reduced expression in adult-treated rats. MacGillivray et al. (2010)[32] reported reduced TPH expression in adult animals directly after acute or chronic FLX treatment, while others failed to see any effect after either 5 or 14 days of FLX treatment [71]. TPH mRNA expression was found to be increased in the DRN after 4 and 8 weeks of FLX treatment [7], but decreases were reported in the medial raphe nucleus, although this was after 2 weeks of treatment and with a much higher dose (i.e., 25 mg/kg vs. 7.5 mg/kg) [72]. Only one study has so far looked into FLX effects during development. Maciag et al. (2006) showed that postnatal exposure (PND 8–21) to FLX was associated with a decrease in TPH immunoreactivity which was maintained into adulthood (PND 130) [33]. So, in both young adult and perinatal animals, chronic FLX treatment decreased TPH expression in the DRN. However, although we also observed TPH expression to be influenced by FLX, we observed a striking dependence of the effect on the age at treatment.

As the developmental regulation of TPH differs between the early postnatal and adolescent period, the lasting effect of FLX may therefore depend on a sensitive time window. In developing rats, TPH activity reaches its peak between 24–30 days after birth and TPH mRNA expression increases until PND 22 after which it decreases to 60% of the initial levels at around adulthood at PND 61 [73]. It might thus be that this normal decrease in TPH during adolescence is counterbalanced by SSRI exposure. As a result, TPH expression could be increased compared to the same-age control animals after drug withdrawal, which would be in line with the ‘use it or lose it’ concept of brain development.

### Implications; relevance for human treatment

The present preliminary observations provide a first step towards an improved understanding of the consequences of FLX exposure on young patient populations. In future studies, it would be interesting to investigate the long-term survival and behavioural effects of the newborn cells. Although the clinical significance of our observations for children and adolescents treated with SSRIs is difficult to predict, it is conceivable that, also in humans, the plasticity of the 5-HT system and of the newborn neurons is higher in children and adolescents than in the mature brain. Pharmacological manipulations of 5-HT would then likely also affect children differently than adults.

### Conclusions

We conclude that the widely prescribed antidepressant FLX has divergent actions on brain measures relevant for depression when comparing adolescent with adult individuals. We have demonstrated that rats treated with FLX during adolescence express a great sensitivity on neurogenesis and 5-HT parameters when measured in adulthood, compared with those treated later, during adulthood. This is highly relevant for future studies investigating the age-dependent differential effects of SSRIs. Our results support the notion that adult neurogenesis is only influenced by SSRI administration during critical developmental time periods. As these drugs, for obvious reasons, have not been thoroughly tested in the adolescent population, the present divergent, age- and brain region- specific results signify continued research is needed into the underlying mechanisms and long-term consequences of juvenile FLX treatment in particular.
