# Correction: Effects of Chronic Fluoxetine Treatment on Neurogenesis and Tryptophan Hydroxylase Expression in Adolescent and Adult Rats

**DOI:** 10.1371/journal.pone.0135876

**Published:** 2015-08-21

**Authors:** Anne Klomp, Lena Václavů, Gideon F. Meerhoff, Liesbeth Reneman, Paul J. Lucassen

There are errors in Fig 3. Please see the complete, correct Fig 3 here.

**Fig 3:**
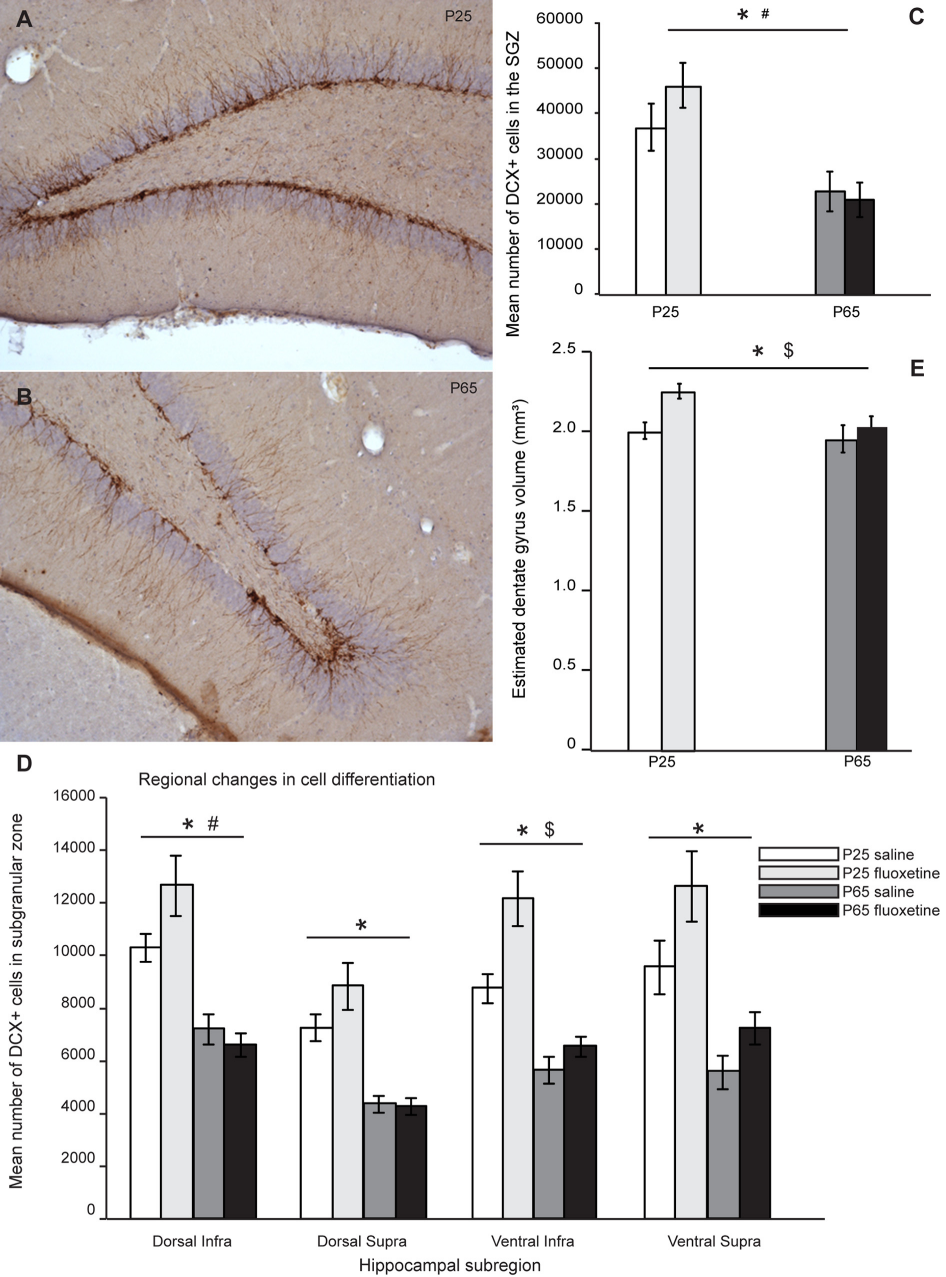
Age-related effects of fluoxetine and regional differences in cell differentiation. An example of doublecortin (DCX) expression along the subgranular zone is shown for adult-treated (A) and adolescent-treated rats (B). C) Two-Way ANOVA revealed both a significant age-by-treatment interaction effect (p = 0.036) and a significant effect of age (p < 0.001) on the expression of DCX+ cells. D) There were regional differences in the amount of DCX+ cells. There was a significant effect of age in all sub-regions (p < 0.001), a significant treatment effect in the ventral infrapyramidal blade of the dentate gyrus (p = 0.021), and a significant age-by-treatment interaction effect in the dorsal infrapyramidal blade (p = 0.028). E) There was both a significant effect of age (p = 0.017) as well as a main effect of treatment (p = 0.045) on dentate gyrus volume. Dentate gyrus volume comprised the SGZ plus GCL. $ = main effect of age; = main effect of treatment; # = age-by-treatment effect. P-values below 0.050 were considered statistically significant. Error bars indicate ± 1 S.E.M.
